# The Negative Interactive Effects of Nostalgia and Loneliness on Affect in Daily Life

**DOI:** 10.3389/fpsyg.2020.02185

**Published:** 2020-09-02

**Authors:** David B. Newman, Matthew E. Sachs

**Affiliations:** ^1^Psychiatry Department, University of California, San Francisco, San Francisco, CA, United States; ^2^Center for Science and Society, Columbia University, New York, NY, United States

**Keywords:** nostalgia, loneliness, affect, well-being, diary

## Abstract

Research has suggested that nostalgia is a mixed, albeit predominantly positive emotion. One proposed function of nostalgia is to attenuate the negative consequences of loneliness. This restorative effect of nostalgia, however, has been demonstrated with cross sectional and experimental methods that lack ecological validity. In studies that have measured nostalgia in daily life, however, nostalgia has been negatively related to well-being. We propose an alternative theory that posits that the effect of nostalgia on well-being depends on the event or experience that elicits nostalgia. We tested this theory by measuring daily states of nostalgia, loneliness, and affect across five daily diary studies (*N* = 504; 6,004 daily reports) that lasted for 14 days. Using multilevel modeling, we found that nostalgia and loneliness were negatively related to positive affect and positively related to negative affect. The negative effects of nostalgia on affective well-being were significantly stronger on days when people felt more lonely as opposed to less lonely. Viewed alternatively, the negative effects of loneliness on affective well-being were stronger on days when people felt more vs. less nostalgic. Thus, in contrast to experimental findings, nostalgia did not attenuate, but rather exaggerated the negative effects of loneliness on affective well-being. These findings support a theoretical account that proposes that the effect of nostalgia on well-being depends on the natural context in which nostalgia is elicited.

## Introduction

“There is no greater sorrow than to recall a happy time when miserable.”

– Dante Alighieri (Inferno, Canto V)

Nostalgia has been defined as a sentimental longing for the past. Poets, novelists, and screenwriters have frequently incorporated this mixed emotion throughout storylines and plots for many years, but a scientific understanding of the nature of nostalgia has only begun to emerge in the past few decades (e.g., Batcho, 2013; Sedikides et al., 2015). This growing body of research has shown that many different situations and settings can trigger feelings of nostalgia, such as adverse weather (van Tilburg et al., 2018), social exclusion (Seehusen et al., 2013), loneliness (Zhou et al., 2008), boredom (van Tilburg et al., 2013), and music (Barrett et al., 2010). Many, although not all, of the triggers of nostalgia are negative experiences.

Although many triggers of nostalgia tend to be negative in nature, the effect of nostalgia on subsequent well-being states appears to be quite positive. For instance, several experiments have shown that nostalgia increases meaning in life, positive affect, self-esteem, and optimism (Wildschut et al., 2006; Routledge et al., 2011; Cheung et al., 2013). In other studies, nostalgia serves a restorative or palliative function by attenuating the negative effects of negative experiences (Sedikides et al., 2015). For example, certain nostalgic feelings can counteract the detrimental effects of meaninglessness (Routledge et al., 2011), they can reduce the deleterious effects of induced self-threat (Vess et al., 2012), and they can increase perceptions of social support following induced loneliness (Zhou et al., 2008). Although a few exceptions to these positive effects of nostalgia have been documented (e.g., Iyer and Jetten, 2011; Verplanken, 2012), the overwhelming number of documented positive effects has led some researchers to conclude that “nostalgia is considered an emotion, and a predominantly positive one at that” (Sedikides et al., 2015).

### Methodological Considerations

When evaluating the functions of nostalgia and its potential benefits on well-being, it is important to consider the methods that have demonstrated these effects. Many of the studies cited above have manipulated nostalgia via the Event Reflection Task, a paradigm that asks participants to write about their *most* nostalgic experience (italics added for emphasis, (Wildschut et al., 2006). Although informative in understanding people’s most nostalgic experience, these manipulations do not capture typical nostalgic feelings that might occur in daily life. It is worth emphasizing that nostalgic feelings that are brought to mind via experimental manipulation are likely quite different from naturally occurring states of nostalgia that are elicited by negative experiences. Hence, experimentally induced nostalgic states likely possess experimental realism, but they lack mundane and psychological realism (Aronson and Carlsmith, 1968; Aronson et al., 1998). Moreover, asking participants to reflect on their most nostalgic experience at one time requires participants to rely on extensive recall, which are often are fraught with biases and heuristics (Bradburn et al., 1987; Schwarz, 2012).

One way of addressing biases inherent in extensive recall tasks is to ask participants about their experiences in daily life in real time or close to real time through the use of daily diary (Bolger et al., 2003) or Ecological Momentary Assessment methods (Shiffman et al., 2008). The goal of daily life methods is to capture a random sample of time points from someone’s life through repeated administration of questions in naturalistic contexts (Newman and Stone, 2019). Studies that have measured fluctuating states of nostalgia in daily life have found that nostalgia looks much more negative than the depiction portrayed by experimental studies (Newman et al., 2020a). Daily and momentary states of nostalgia were negatively related to well-being, and these negative relationships remained after controlling for the effects of negative experiences on well-being. Lagged analyses from one day to the next showed that nostalgia predicted increases in rumination and sadness and decreases in feelings of peacefulness and calm (Newman et al., 2020a, Study 3).

In an attempt to reconcile the differences between the negative relationships between nostalgia and well-being in daily life, Newman et al. (2020a) asked participants to write about their most nostalgic experience (following the instructions of the Event Reflection Task) at one time and to write about their daily nostalgic feelings each day for one week. If they did not feel nostalgic on a particular day, they were asked to write about an ordinary experience. After each writing exercise, participants rated how positive and how negative each experience was. They found that people’s most nostalgic experiences were more positive and less negative than their daily nostalgic feelings (Study 5). In fact, daily nostalgic feelings were similar in valence to ordinary, non-nostalgic experiences in daily life. The conclusion from these studies was that the deliberate engagement in the recollection of extremely nostalgic moments may improve well-being, but involuntarily experiencing nostalgia elicited by situational cues may not feel particularly good.

### An Alternative Perspective

The findings from studies that have measured nostalgia in daily life provide a framework to understand nostalgia in ecologically valid contexts. This has led to a theoretical perspective that incorporates the nature of the daily situations and experiences that elicit feelings of nostalgia and their downstream consequences. According to this theory, the valence of the nostalgia-eliciting event will influence the valence of the nostalgic feeling. As demonstrated by Newman et al. (Study 5), some feelings of nostalgia were considerably more positive than others. In other words, some nostalgic feelings are very sweet with just a slight tinge of bitterness, whereas other nostalgic feelings are very bitter with just a tinge of sweetness. A positive event, such as spending time with childhood friends, will elicit more highly positively-valenced nostalgic feelings, whereas negative experiences, such as social isolation, will elicit more negatively-valenced nostalgic feelings.

Subsequently, the valence of the nostalgic feeling will influence well-being. Nostalgic feelings that are more positively-valenced will lead to an increase in well-being, whereas nostalgic feelings that are more negatively-valenced will lead to a decrease in well-being. The theory makes the assumption that the nostalgic memory is used to form a representation of the target judgment (e.g., how my life is currently going), resulting in an assimilation (Bless and Schwarz, 2010)^1^. This theory predicts that nostalgia will exaggerate (as opposed to buffer) the effect of the nostalgia-eliciting experience on well-being.

### Present Research

The present research considered the interaction effect of loneliness and nostalgia on affective well-being. Nostalgia has been shown to buffer the negative effects of loneliness (Zhou et al., 2008), but these studies relied on cross-sectional data that do not capture within-person variation in daily life and experiments which lack ecological validity. The present studies addressed these limitations by measuring daily states of nostalgia in several daily diary studies. We hypothesize that the negative effects of nostalgia on affective well-being will be amplified on days when people feel lonely. Because feelings of loneliness should lead to nostalgic feelings that are more negative than positive, these nostalgic experiences should consequently have a negative influence on affective well-being. This prediction contrasts with those derived from experimental studies that suggest that nostalgia can buffer or attenuate the negative effects of loneliness.

## Methods

### Participants and Procedure

Participants were undergraduate students at a large university who received research credit in exchange for their participation. After signing up for the study, they either watched a short instructional video or participated in a video chat with one of the researchers to learn about the procedure. Over the course of two weeks, the participants received an email each evening at 9:00 pm with a link to a questionnaire. They were instructed to complete the questionnaire at the end of their day just before going to bed. A reminder email was sent on the following morning at 7:00 am and responses were accepted until 10:00 am.

Daily reports were removed from final analyses if they were completed after 10:00 am, if they were completed in less than 2 or 3 min (depending on the number of questions asked in each sample), if multiple entries were completed on the same day, or if an instructed response item (e.g., “Please select ‘occurred and not important’ for this question,” as recommended by Meade and Craig, 2012) was answered incorrectly. Additionally, participants who completed less than five valid daily responses were removed. In total, participants completed 6,541 daily reports. After data cleaning, we analyzed 6,016 daily reports (91.97%) from a total of 504 participants (*M*_age_ = 20.10; *SD* = 1.89; 78.2% female). On average, participants completed 11.94 daily reports (*SD* = 2.18, median = 13) indicating good compliance (see Nezlek, 2012, for a discussion of typical compliance rates in daily diary studies).

Data from five different diary studies were compiled because their procedures and measures were similar. Some of the results we report here are secondary analyses from published data that was used for different purposes than the present paper (Newman et al., 2020a,b).

### Measures

Nostalgia was measured with the 4-item Personal Inventory of Nostalgic Experiences (PINE) scale (Newman et al., 2020a). This scale has been validated in previous research and has demonstrated better psychometric properties than the commonly used Southampton Nostalgia Scale (SNS; Routledge et al., 2008; Barrett et al., 2010). As is common practice in daily diary studies, the items were reworded slightly to be appropriate for daily administration. An example of one of the items is “How nostalgic did you feel today?” Responses were recorded on a 7-point scale (1 = *not at all*, 7 = *very much*).

Loneliness and affect were measured in a similar manner. Affect was conceptualized using the affective circumplex model which distinguishes valence and arousal (Barrett and Russell, 1998). We used five adjectives to measure each quadrant of the circumplex. Positive activated (PA) affect was measured with happy, excited, enthusiastic, delighted, and glad; positive deactivated (PD) affect was measured with calm, peaceful, relaxed, at ease, and contented; negative activated (NA) affect was measured with stressed, tense, nervous, angry, and annoyed; and negative deactivated (ND) affect was measured with sad, gloomy, miserable, depressed, and disappointed. Following similar methods used by Jonason et al. (2008) and Doane and Adam (2010), we measured loneliness with the items alone and lonely. For each loneliness and affect adjective, participants were asked to report how strongly they felt each one today. Responses were recorded on a 7 point scale (1 = *did not feel this way at all*, 4 = *felt this way moderately*, 7 = *felt this way very strongly*).

## Results

### Descriptive Statistics

Because the data were hierarchical in nature, multilevel modeling was used for all analyses. The five studies presumably represented a sample of time points from the larger hypothetical population of all times; we therefore conceptualized the structure as a three-level model in which days were nested within persons, and persons were nested within study. We used the program HLM 7 (Raudenbush et al., 2011) and present unstandardized coefficients.

To provide estimates of the means and variances of each measure, we conducted unconditional models in which no predictors were entered at any level. These models indicated that there was more within-person variance than between-person variance for each variable (see Table 1). There was very little between-study variance. Reliabilities were calculated in a manner described by Nezlek (2017), and each construct was measured reliably.

**TABLE 1:** Descriptive statistics.

		**Variance**			
**Variable**	**Mean**	**Within-person**	**Between-person**	**Between-study**	**Reliability**
Nostalgia	2.67	1.62	1.43	0.02	0.90
Loneliness	2.27	1.32	1.21	0.00	0.82
Positive activated affect	3.74	1.31	1.10	0.00	0.84
Positive deactivated affect	3.59	1.10	0.95	0.00	0.83
Negative activated affect	3.05	1.17	0.74	0.00	0.63
Negative deactivated affect	2.31	1.09	0.84	0.01	0.80

### Primary Analyses

First, we examined the within-person relationships between nostalgia and affective states. In separate models, affect was entered as the outcome variable and nostalgia was entered as the sole level-1 predictor, centered around each individual’s mean as follows:


$$\mathrm{Day}\,\mathrm{level}:y_{i\,j\,k}\,\left(\mathrm{affect}\right)=\pi_{0\,j\,k}+\pi_{1\,j\,k}\,\left(\mathrm{nostalgia}\right)+e_{i\,j\,k}$$

$$\mathrm{Person}\,\mathrm{level}:\pi_{0\,j\,k}=\beta_{00\,k}+r_{0\,j\,k}\,\pi_{1\,j\,k}=\beta_{10\,k}+r_{1\,j\,k}$$

$$\mathrm{Study}\,\mathrm{level}:\beta_{00\,k}=\gamma_{000}+u_{00\,k}\,\beta_{10\,k}=\gamma_{100}+u_{10\,k}$$

Error terms were trimmed if their *p*-values exceeded 0.15 as recommended by Nezlek (2012)^2^. These models showed that nostalgia was negatively related to PA, *b* = −0.03, *t* = 2.24, *p* = 0.025, and PD, *b* = −0.05, *t* = 3.34, *p* < 0.001, and was positively related to NA, *b* = 0.13, *t* = 11.70, *p* < 0.001, and ND, *b* = 0.19, *t* = 7.17, *p* < 0.001. Consistent with the findings by Newman et al. (2020a), nostalgia was negatively related to daily affective well-being.

Next, we examined the within-person relationships between loneliness and affective states and nostalgia in a similar manner. Loneliness was negatively related to PA, *b* = −0.22, *t* = 12.73, *p* < 0.001, and PD, *b* = −0.18, *t* = 11.61, *p* < 0.001, and was positively related to NA, *b* = 0.29, *t* = 23.62, *p* < 0.001, and ND, *b* = 0.50, *t* = 49.24, *p* < 0.001. Loneliness was positively related to nostalgia, *b* = 18, *t* = 39.15, *p* < 0.001. In sum, loneliness and nostalgia were negatively related to affective well-being.

Critical to the main hypothesis, we examined whether the negative effects of nostalgia on affect were moderated by daily states of loneliness. To do so, we group-mean centered (i.e., centered around each individual’s mean) nostalgia and loneliness and multiplied these variables together to create an interaction term at level 1. In separate models, affective states were entered as the outcome measures, nostalgia and loneliness were group-mean centered, and the interaction term was entered uncentered at level 1. No predictors were added at levels 2 or 3.


$$\mathrm{Day}\,\mathrm{level}:\;\,y_{\text{ijk}}\,\left(\mathrm{affect}\right)=\pi_{0\,j\,k}+\pi_{1\,j\,k}\,\left(\mathrm{loneliness}\right)\,\;$$

$$+\pi_{2\,j\,k}\,\left(\mathrm{nostalgia}\right)+\pi_{3\,j\,k}\,\left(\mathrm{interaction}\right)+e_{\text{ijk}}$$

These models indicated that the within-person relationships between nostalgia and affective states were moderated by daily states of loneliness (see Table 2). To understand the nature of these interactions, we calculated estimates of the affective states on days that were one standard deviation above and below the mean for nostalgia and loneliness. As described by Nezlek (2011), the standard deviations were taken from unconditional models for each respective variable. These estimates are provided in the top portion of Table 3. The effect of nostalgia on PA and PD was slightly positive on days (i.e., PA and PD increased) when people felt lower levels of loneliness, and the effect of nostalgia on PA and PD was slightly negative (i.e., PA and PD decreased) on days when people felt higher levels of loneliness. The positive effects of nostalgia on NA and ND (i.e., NA and ND increased) were stronger on days that were high in perceived loneliness. In sum, the negative effects of nostalgia on affective well-being were stronger on days when people felt quite lonely vs. less lonely.

**TABLE 2:** Parameter estimates of all variables in interaction models.

	**Loneliness coefficient**			**Nostalgia coefficient**			**Interaction coefficient**		
**DV**	***b***	***t***	***p***	***b***	***t***	***p***	***b***	***t***	***p***
PA	−0.21	29.24	<0.001	0.03	1.52	0.202	−0.03	2.97	0.003
PD	−0.17	20.11	<0.001	−0.01	0.80	0.423	−0.03	2.50	0.012
NA	0.27	14.10	<0.001	0.07	4.18	0.014	0.02	3.92	<0.001
ND	0.44	24.66	<0.001	0.10	4.32	0.012	0.03	7.03	<0.001

*DV, dependent variable; PA, positive activated affect; PD, positive deactivated affect; NA, negative activated affect; ND, negative deactivated affect.*

**TABLE 3:** Estimates of affect at one standard deviation above and below means of nostalgia and loneliness in interaction models.

**Interpretation: The Effect of Nostalgia on Well-Being is Moderated by Loneliness**						
	**Low Loneliness (-1 *SD*)**			**High Loneliness (+ 1 *SD*)**		
		
**DV**	**-1 *SD* Nostalgia**	**+ 1 *SD* Nostalgia**	**High vs. Low Nostalgia**	**-1 *SD* Nostalgia**	**+ 1 *SD* Nostalgia**	**High vs. Low Nostalgia**
PA	3.91	4.06	0.15	3.50	3.49	−0.01
PD	3.76	3.81	0.05	3.45	3.35	−0.10
NA	2.68	2.82	0.14	3.23	3.47	0.24
ND	1.71	1.89	0.18	2.62	2.99	0.37
**Interpretation: The Effect of Loneliness on Well-Being is Moderated by Nostalgia**						
	**Low Nostalgia (**-**1 *SD*)**			**High Nostalgia (+ 1 *SD*)**		
**DV**	**-1 *SD L*oneliness**	**+ 1 *SD* Loneliness**	**High vs. Low Loneliness**	**-1 *SD* Loneliness**	**+ 1 *SD* Loneliness**	**High vs. Low Loneliness**

PA	3.91	3.50	−0.41	4.06	3.49	−0.57
PD	3.76	3.45	−0.31	3.81	3.35	−0.46
NA	2.68	3.23	0.55	2.82	3.47	0.66
ND	1.71	2.62	0.91	1.89	2.99	1.10

*DV, dependent variable; PA, positive activated affect; PD, positive deactivated affect; NA, negative activated affect; ND, negative deactivated affect.*

These effects could also be interpreted in terms of the moderation of daily states of nostalgia on the within-person relationships between loneliness and affective well-being (see the bottom of Table 3). Framed in this manner, loneliness was more strongly negatively related to PA and PD and was more strongly positively related to NA and ND on days when people felt more nostalgic than on days when they felt less nostalgic. For example, consider the interaction effect involving ND. On days low in nostalgia, the effect of loneliness on ND was 0.91. On days high in nostalgia, the effect was 1.10. Thus, nostalgia exaggerated or amplified the negative effects of loneliness on affective well-being.

### Suppression Analyses

The analyses described above examined the interaction effects of nostalgia and loneliness on affective well-being states. In previous research, the buffering effect of nostalgia has been tested through inconsistent mediation, also known as statistical suppression (MacKinnon et al., 2000; Zhou et al., 2008). In these models, the negative effects of loneliness have been suppressed by nostalgia. For example, after adding nostalgia as a predictor in a model, Zhou et al. (2008) found that the negative relationship between loneliness and perceived social support was strengthened in magnitude, i.e., became more negative.

We aimed to examine the possibility that the negative effects of loneliness on well-being would be suppressed by daily states of nostalgia. To do so, we created three models. The first model examined the effect of loneliness on affective well-being (i.e., the total effect). The second model examined the effect of loneliness on nostalgia (i.e., the a-path), and the third model included loneliness and nostalgia as predictors of affective well-being, which examines the b-path and the direct effect. We calculated the within-person indirect effect from a multilevel structural equation model using the program MPlus Version 8.4 (Muthén and Muthén, 1998–2017) (see Preacher et al., 2010, for a description)^3^.

These models showed that the within-person indirect effects were significant or marginally significant for PD, NA, and ND (see Table 4). Contrary to findings from previous research, the effects of loneliness on affective well-being did not become stronger after including nostalgia as a mediator. That is, nostalgia did not buffer against the negative effects of loneliness on affective well-being. Rather, nostalgia served as a mediator and helped explain why loneliness was negatively related to affective well-being. These findings are consistent with the moderation analyses described above that paint nostalgia in a more negative light.

**TABLE 4:** Parameter estimates from mediation models.

	**a-path**			**b-path**			**Total effect (c)**			**Direct effect (c’)**			**Indirect effect**		
**DV**	***b***	***t***	***p***	***b***	***t***	***p***	***b***	***t***	***p***	***b***	***t***	***p***	***b***	***95% CI***	***p***
PA	0.22	10.90	<0.001	0.02	1.61	0.107	−0.22	12.73	<0.001	−0.22	12.68	<0.001	−0.01	[−0.02, 0.00]	0.127
PD	0.22	10.90	<0.001	−0.01	0.98	0.328	−0.18	11.61	<0.001	−0.17	11.24	<0.001	−0.01	[−0.02, 0.00]	0.058
NA	0.22	10.90	<0.001	0.08	5.44	<0.001	0.29	17.63	<0.001	0.27	16.34	<0.001	0.02	[0.01, 0.03]	0.001
ND	0.22	10.90	<0.001	0.11	8.11	<0.001	0.48	29.23	<0.001	0.45	28.06	<0.001	0.03	[0.02, 0.04]	<0.001

*The a-path refers to the effect of loneliness on nostalgia; the b-path refers to the effect of nostalgia on affect; the total effect refers to the effect of loneliness on affect without controlling for nostalgia; the direct effect refers to the effect of loneliness on affect after controlling for nostalgia; the indirect effect refers to the effect of loneliness on affect via nostalgia.*

## Discussion

Across five daily diary studies, feelings of nostalgia varied considerably from one day to the next. Replicating previous findings at a within-person level of analysis, daily feelings of nostalgia were negatively related to affective well-being. The present findings advance our understanding of these within-person relationships by showing that the negative effects of nostalgia on affective well-being were stronger on days when people felt higher levels of loneliness than on days when they felt lower levels of loneliness. These findings are consistent with the notion that nostalgic feelings may be influenced by various contextual factors that vary from one day to the next. When the nostalgia-eliciting event or experience is a negative one (e.g., feeling lonely), the nostalgic feeling will be tinged with more sadness than it otherwise would be if the nostalgia-eliciting event were a more positive one (e.g., socializing with close friends). The valence of the nostalgic feeling will subsequently influence well-being states.

We also found no evidence that nostalgia attenuates or buffers the negative effects of loneliness on affective well-being states. That is, the negative effects of loneliness on affect were not suppressed by daily nostalgic feelings. Rather, nostalgia actually mediated these relationships. The effects of loneliness on affect were reduced in magnitude after entering nostalgia as a mediator. Thus, the reason people do not feel well when they feel lonely can be attributed in part to feelings of nostalgia.

### Reemphasis of Methodological and Conceptual Considerations

These findings add to a small but growing body of research that shows that nostalgia experienced in daily life does not look quite as rosy as the view of nostalgia proposed from experimental and cross-sectional studies. For example, in a recent experience sampling study of employees, momentary states of nostalgia were not significantly related to momentary states of positive affect (van Dijke et al., 2019). Our findings also dovetail nicely with those reported by Muise et al. (2020) who measured daily states of sexual nostalgia, defined as reflections of positive sexual experiences with former romantic partners. They found that people were more likely to engage in sexual nostalgic thoughts on days when they were less satisfied with their current romantic relationship and that individual differences in sexual nostalgia, measured as an aggregation of daily states of sexual nostalgia, were negatively related to sexual and relationship satisfaction 3 months later.

When considering the discrepancies between the experimental findings that posit that nostalgia is a “predominantly positive emotion” (Sedikides et al., 2015) and daily life methods that suggest that nostalgia is negatively related to well-being (Newman et al., 2020a), it is important to remember that different methods address different questions and have unique strengths and weaknesses. Stated more pessimistically, “all research strategies and methods are seriously flawed” (McGrath, 1982, p. 70). Experimental studies can address causal effects that may exist, but they lack ecological validity and do not provide any information about how frequently nostalgia occurs or how it relates to other variables in daily life. Daily life methods address these limitations quite well, but they obviously cannot make firm causal claims because nostalgic states are measured rather than manipulated.

Additionally, many experiments have used between-subject designs, whereas our studies examined within-person processes. These unique levels of analysis are mathematically orthogonal (Nezlek, 2001) and may represent distinct psychological processes (Affleck et al., 1999). Moreover, several studies that have found support for the buffering effect of nostalgia have measured nostalgia with the Southampton Nostalgia Scale (e.g., Zhou et al., 2008; Juhl et al., 2010), which combines nostalgia proneness with valuing nostalgia. We measured nostalgia in daily life with the Personal Inventory of Nostalgic Experiences (PINE) scale, a scale that reliably captures nostalgic feelings. Previous research has shown that the PINE scale is more negatively related to well-being than the SNS (Newman et al., 2020a, Study 2).

In addition to these methodological differences, it is important to highlight a conceptual similarity between our studies and prior experimental research. Similar to prior research, we define nostalgia as a sentimental longing for the past. People’s recall of their most nostalgic feeling and daily nostalgic feelings are all instances of nostalgia. Thus, nostalgia measured in daily life is not an entirely new construct from the one that is captured in experiments. They simply differ in their prototypicality and in how they are elicited.

### Qualifications and Future Research

With these methodological differences in mind, it is important to qualify some of our results and distinguish what we can state with empirical conviction from what we surmise. Because daily states of all variables were assessed at one time at the end of each day, we do not know the exact sequence of the temporal states that occurred within each day. Additionally, we cannot make firm causal statements due to potential third variable confounds. It is possible that loneliness and affect interact to influence nostalgia, namely that people would be most likely to feel nostalgic when they feel lonely and sad. This possibility seems unlikely, however, given that lagged analyses from one day to the next showed that nostalgia actually increased NA and ND and decreased PD on the following day. Nevertheless, future research is needed to examine the specific sequence of feelings and states that occur within a day.

It is also important to stress that our findings demonstrate what typically happens in daily life. It is possible that nostalgia may attenuate the negative effects of loneliness and other negative experiences in certain situations. For instance, consider a situation in which loneliness increases nostalgia and thereby leads one to view pictures of old friends on social media. If that particular act causes one to talk with the old friends, that would certainly help buffer against the initial negative effects of feeling lonely. Our findings from daily diaries, however, showed that this hypothetical example likely does not occur very frequently.

Our outcome measures were affective well-being states, and we should be cautious in generalizing our findings beyond affective well-being. Many of the experimental studies have used a range of outcome measures, including vitality (Routledge et al., 2011), organizational citizenship behavior (van Dijke et al., 2015), and collective guilt (Baldwin et al., 2018). Future research is needed to examine differences between the effect of daily nostalgic feelings and experimentally induced nostalgia on these outcomes.

## Conclusion

In contrast to previous research that has relied on experimental manipulations and cross-sectional designs, we found that nostalgia did not buffer against the negative effects of loneliness. Instead, using daily diary methods to measure natural fluctuations of nostalgia, we found that the negative effects of nostalgia were amplified on days when people felt lonely. Moreover, the negative effect of loneliness on affective well-being states were not attenuated by nostalgia, but rather, were mediated or explained by daily nostalgic feelings. Although the deliberate engagement in atypically positive nostalgic experiences induced via experimental manipulation may lead to beneficial effects, ordinary nostalgic feelings experienced in the natural contexts of daily life appear to be less beneficial.

## Data Availability Statement

The data are stored at osf.io/dtw5m and are available by request from the corresponding author.

## Ethics Statement

The studies involving human participants were reviewed and approved by the Institutional Review Board University of Southern California. The patients/participants provided their written informed consent to participate in this study.

## Author Contributions

DN and MS discussed the theory and hypotheses, contributed to manuscript revisions, and read and approved the submitted version. DN collected, organized, analyzed the data, and wrote the first draft of the manuscript. Both authors contributed to the article and approved the submitted version.

## Conflict of Interest

The authors declare that the research was conducted in the absence of any commercial or financial relationships that could be construed as a potential conflict of interest.

## Footnotes

1 It is possible for a nostalgic reflection to result in a contrast effect if the information brought to mind is used to form a representation of the standard (i.e., how my life was in the past) against which the target is compared (i.e., how my life is currently). In this manuscript, we consider assimilation effects only for simplicity.

2 In a few of the models, error terms at level 3 did not reach this level of significance, so we were more lenient to allow these models to converge with at least one random effect at this level.

3 Due to complexities of testing mediation/suppression in three-level models, we opted instead to run these models using two-level models with days nested within-persons (Preacher, 2011).
