# Corrigendum: The negative interactive effects of nostalgia and loneliness on affect in daily life

**DOI:** 10.3389/fpsyg.2022.1046621

**Published:** 2022-10-06

**Authors:** David B. Newman, Matthew E. Sachs

**Affiliations:** ^1^Psychiatry Department, University of California, San Francisco, San Francisco, CA, United States; ^2^Center for Science and Society, Columbia University, New York, NY, United States

**Keywords:** nostalgia, loneliness, affect, well-being, diary

In the published article, a citation for Newman et al. (2020a) was missing. The citation has now been inserted in **Methods**, “*Participants and Procedure*,” paragraph 3 and should read:

“Some of the results we report here are secondary analyses from published data that was used for different purposes than the present paper (Newman et al., 2020a,b).”

The authors apologize for this error and state that this does not change the scientific conclusions of the article in any way. The original article has been updated.

## Publisher's note

All claims expressed in this article are solely those of the authors and do not necessarily represent those of their affiliated organizations, or those of the publisher, the editors and the reviewers. Any product that may be evaluated in this article, or claim that may be made by its manufacturer, is not guaranteed or endorsed by the publisher.

