# Supplementary material for: The Negative Interactive Effects of Nostalgia and Loneliness on Affect in Daily Life
Source: Front Psychol. 2020 Sep 2;11:2185. doi: 10.3389/fpsyg.2020.02185 (PMC7492671; doi:10.3389/fpsyg.2020.02185)
Supplement: Supplementary file 1 [file Table_1.docx]

Please indicate the extent to which each of the following statements describe you today.

|  | Not at all |  |  |  |  |  | Very much |
| --- | --- | --- | --- | --- | --- | --- | --- |
| How nostalgic did you feel today? |  |  |  |  |  |  |  |
| To what extent did you feel sentimental for the past? |  |  |  |  |  |  |  |
| How much did you feel a wistful affection for the past? |  |  |  |  |  |  |  |
| To what extent did you feel a longing to return to a former time in your life? |  |  |  |  |  |  |  |

Listed below are a number of feelings and emotions. Please indicate how strongly you felt that way today.

|  | did not feel this way at all |  |  | felt this way moderately |  |  | felt this way very strongly |
| --- | --- | --- | --- | --- | --- | --- | --- |
| enthusiastic |  |  |  |  |  |  |  |
| calm |  |  |  |  |  |  |  |
| stressed |  |  |  |  |  |  |  |
| depressed |  |  |  |  |  |  |  |
| delighted |  |  |  |  |  |  |  |
| peaceful |  |  |  |  |  |  |  |
| angry |  |  |  |  |  |  |  |
| disappointed |  |  |  |  |  |  |  |
| happy |  |  |  |  |  |  |  |
| relaxed |  |  |  |  |  |  |  |
| annoyed |  |  |  |  |  |  |  |
| miserable |  |  |  |  |  |  |  |
| glad |  |  |  |  |  |  |  |
| contented |  |  |  |  |  |  |  |
| tense |  |  |  |  |  |  |  |
| gloomy |  |  |  |  |  |  |  |
| at ease |  |  |  |  |  |  |  |
| excited |  |  |  |  |  |  |  |
| nervous |  |  |  |  |  |  |  |
| sad |  |  |  |  |  |  |  |
| alone |  |  |  |  |  |  |  |
| lonely |  |  |  |  |  |  |  |
